# Optimization of the Warpage of Fused Deposition Modeling Parts Using Finite Element Method

**DOI:** 10.3390/polym13213849

**Published:** 2021-11-08

**Authors:** Daniyar Syrlybayev, Beibit Zharylkassyn, Aidana Seisekulova, Asma Perveen, Didier Talamona

**Affiliations:** Department of Mechanical and Aerospace Engineering, School of Engineering & Digital Sciences, Nazarbayev University, Nur-Sultan 010000, Kazakhstan; daniyar.syrlybayev@nu.edu.kz (D.S.); beibit.zharylkassyn@nu.edu.kz (B.Z.); aidana.seisekulova@nu.edu.kz (A.S.); asma.perveen@nu.edu.kz (A.P.)

**Keywords:** warpage, finite element analysis (fem), FDM, Taguchi, multilateral

## Abstract

Fused deposition modeling (FDM) is one of the most affordable and widespread additive manufacturing (AM) technologies. Despite its simplistic implementation, the physics behind this FDM process is very complex and involves rapid heating and cooling of the polymer feedstock. As a result, highly non-uniform internal stresses develop within the part, which can cause warpage deformation. The severity of the warpage is highly dependent on the process parameters involved, and therefore, currently extensive experimental studies are ongoing to assess their influence on the final accuracy of the part. In this study, a thermomechanical Finite Element model of the 3D printing process was developed using ANSYS. This model was compared against experimental results and several other analytical models available in the literature. The developed Finite Element Analysis (FEA) model demonstrated a good qualitative and quantitative correlation with the experimental results. An L9 orthogonal array, from Taguchi Design of Experiments, was used for the optimization of the warpage based on experimental results and numerical simulations. The optimum process parameters were identified for each objective and parts were printed using these process parameters. Both parts showed an approximately equal warpage value of 320 μm, which was the lowest among all 10 runs of the L9 array. Additionally, this model is extended to predict the warpage of FDM printed multi-material parts. The relative percentage error between the numerical and experimental warpage results for alternating and sandwich specimens are found to be 1.4% and 9.5%, respectively.

## 1. Introduction

Fused deposition modeling is one of the Additive Manufacturing (AM) processes in which the part is manufactured layer by layer from the thermoplastic polymers extruded through a heated nozzle, which moves along the programmed path. It was originally developed by Stratasys Inc., and nowadays has become one of the most popular and affordable AM processes [1]. One of the most significant advantages of the FDM process is its ability to produce parts of complex shapes [2]. In addition, the FDM process requires no tooling [3] and offers a high degree of customization, as the cost per part produced by AM is lower for small batches [4]. Nonetheless, several drawbacks limit its use in the industry, and the most important among them are build speed, mechanical properties, and part dimensional accuracy [5,6].

The accuracy of the parts produced by FDM is highly dependent on the process parameters employed. For this reason, recently there have been several studies conducted to optimize the quality of the end-product produced by FDM. An approach involving benchmark artifacts was also used in several studies to compare the accuracy of the FDM with other popular AM processes [7,8]. In addition, Mahmood et al. (2018) [9] performed Taguchi optimization of the 13 common printing parameters to achieve the highest accuracy of the features of the benchmark artifact. Anitha et al. [10] used Taguchi optimization to study how surface roughness is affected by printing parameters. It was found that layer thickness is the most significant parameter while printing speed is the least significant one. Similar conclusions were obtained by [11,12]. Multi-objective Grey Taguchi optimization of the FDM was performed by Sood et al. [3] to minimize length, width, and thickness deviations. It was found that shrinkage is predominant along the length and width. This occurs during the cooling from the glass transition temperature of the plastic to room temperature. However, thickness deviations are always positive. One of the reasons for this is the positive round-off error of the printer.

Apart from the dimensional deviations, the warpage of the parts is also a serious issue in FDM. Due to rapid cooling and heating during the deposition process, non-uniform shrinkage occurs within a part, and it starts to warp. Several studies were performed to investigate how the warpage is affected by printing parameters. Experiments show that warpage is highly affected by the layer thickness, and the lower the layer thickness, the higher the warpage [13,14,15,16]. On the other hand, several analytical models in which an elastic material behavior was assumed [15,17,18,19] showed the direct correlation between the layer thickness and the warpage. Similarly, Armilota et al. [20] developed an analytical model, which considers reheating of the layers and yielding. This model showed a greater accuracy compared to the simple models based on the theory of elasticity.

The reasons for the discrepancy between the analytical models and experiments are still unknown and under investigation. Finite Element Analysis (FEA) of the FDM process can be used to understand these discrepancies. Recent studies [21,22,23,24] have shown that coupled thermomechanical studies using FEA can be successfully employed to recreate the thermal history of the part and model its residual stresses and deformations. It was found that residual stresses are highly affected by the rate of cooling and increasing the convection will increase the development of residual stresses, which leads to excessive distortions and delamination. Cattenone et al. [25] studied how the Finite Element setup affects the result for distortions and residual stresses. Distortions of the semi-crystalline polymers were studied by Samy et al. [26], who found a direct correlation among warpage, residual stresses, and relative crystallization. However, works mentioned above consider simple-shaped bodies, which can be meshed by the structured grid. Several studies were also employed to model complex shapes [27,28,29,30]. An approach used in these studies is to approximate geometry around the boundary by voxelization.

As discussed, warpage was studied in numerous works previously, however, for now, the results are inconsistent. This is especially true for the layer thickness. In addition, although the effect of the cooling rate on the warpage is known, to the authors’ knowledge no study attempted to consider the effects of the nozzle and build-plate temperatures. Furthermore, FEA has already shown its reliability in modeling the FDM printing process. It allows obtaining and assessing data that cannot be measured during the experiments and provides a better insight into the warpage occurrence. However, such nonlinear, transient simulations require large computational power. Hence, the main objective of this work was to develop a transient thermomechanical, simple material, model using FEM. This model was used to optimize and study the effect of three parameters such as layer thickness, bed temperate and nozzle temperature. FEA results were validated against several analytical models and experimental results. In a second phase, this FEA model was extended to multi-material FDM printing.

## 2. Methodology

The Finite Element Model of the FDM process was built and used to predict and optimize warpage. The results were verified against experimental results and analytical models available in the literature. The following sections described the design of experiment (DOE) of the procedure involved in every step of the investigation.

### 2.1. Finite Element Model

**Figure 1:**
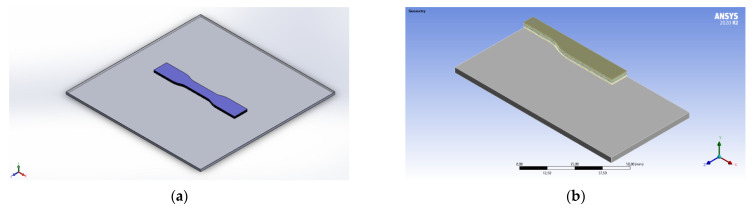
Problem domain (**a**) tensile test sample (**b**) quarter symmetry.

The model selected for Finite Element Analysis and 3D printing is the standard sample for tensile testing along with the build platform, as shown in Figure 1a. The build platform having dimensions equal to those in the actual printer was added to the analysis to represent the heat transfer through the bottom layer more accurately. The part was selected as it is long and thin, which allows obtaining larger warpage and facilitates the measurements.

To simplify the analysis, the following assumptions were used:

(1) The phase change and creep effects at high temperatures were neglected. This is a common assumption, which was employed in several previous studies [21,25] and did not show any significant deviations.
(2) It was assumed that the entire layer is deposited at once (or instantaneously). This assumption is also commonly used in analytical models [15,17,18,19,20]. The results from El Moumen et al. [23] also show that this assumption does not cause significant deviations when deformations are modeled using FEA.
(3) Plastic was assumed to have isotropic material properties with flawless microstructure.
(4) Chamber and plate temperatures were assumed to have constant temperatures, and natural convection effects were neglected.

The assumptions (3) and (4) were also successfully employed in previous studies [21,25] and did not lead to significant errors between experimental and numerical results.

Due to the second assumption, the printed part is symmetrical and only one-quarter of the part needs to be modeled, with proper symmetry conditions to be applied at the boundaries. This reduced the computational time of the analysis significantly. The final domain used for Finite Element simulations is shown in Figure 1b.

**Figure 2:**
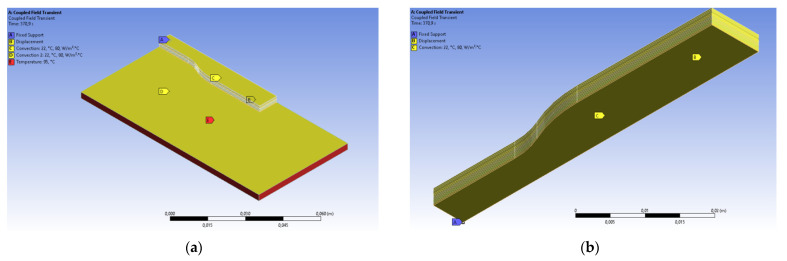
(**a**) Thermal boundary conditions. (**b**) Structural boundary conditions (zero translation along the normal direction and rotation on each node of symmetry surfaces).

During the FDM printing process, the plastic filaments are heated and extruded through a nozzle. Upon cooling, the strains and internal stresses start to develop within the part. For this reason, in the following study, the thermal history of the built part was re-created. The equation governing the thermal analysis is given as follows:

(1) $$c\frac{\partial T}{\partial t}=\frac{\partial }{\partial x_{i}}\left(k\frac{\partial T}{\partial x_{j}}\right)+q\;$$

with boundary conditions $T=T_{b}$ on $\Gamma_{u}$ and $-\frac{\partial T}{\partial x_{i}}n_{i}=q_{n}$ on $\Gamma_{j}$. The initial condition is given by $T\left(x,\;y,\;z,\;0\right)=T_{0}.$ Here, *T* is temperature, *k* is heat conductivity, *c*—specific heat, and *q*—body heat per unit volume (zero in this study), $q_{n}$—heat transfer rate at the boundary per unit area, $T_{b}$—bed temperature, $\Gamma_{u}$—Dirichlet boundary, $\Gamma_{j}$—Neumann boundary, and $T_{0}$—initial temperature. The initial temperature was assumed to be the temperature of the nozzle used in real printing. The Dirichlet boundary in the following analysis was imposed on the whole build plate, as shown in Figure 2a. The Neumann boundary was set on the whole surface of the part, including the top surface of the platform.

In the following study, the heat transfer at the boundary might occur due to convection and radiation. Convective heat transfer $q_{c}\;$ can be found by the following equation.

(2) $$q_{c}=h\left(T-T_{c}\right)$$

where h is the convective heat transfer coefficient and $T_{c}$ is the temperature of the surroundings. Because the printer used for the experiments is open, it was assumed that $T_{c}$ is constant and equal to 22 °C (room temperature). The convective heat transfer coefficient can be found using an empirical relation given as follows:

(3) $$Nu_{L}=\frac{hL}{k_{air}}=\left(0.037Re_{L}-871\right)\sqrt[{3}]{Pr}\;$$

where $Re_{L}$ and Pr are the Reynolds and Prandtl numbers of the air around the part [31]. Using this relation, convective heat transfer was calculated, and it is equal to 80 $W/m^{2}C$. This is consistent with the values commonly found in the literature [21,22,27]. Usually, heat radiation from the part surface is very small and was ignored in the previous studies [21,25]. As it was suggested by Costa et al. [32], radiation heat transfer can be neglected when the convective loss is large (larger than 60 $W/m^{2}C$). Hence in this work, radiation was ignored.

The solution of Equation (1) was used to find the strain field using the following equation.

(4) $$\varepsilon_{ij}^{t}=\alpha I\left(T-T_{c}\right)\;$$

where $\varepsilon_{ij}^{t}$ is a thermal strain and α is the linear heat expansion coefficient, *I*—identity matrix. The result of the thermal strain was used as a boundary condition for the structural analysis. This is governed by the equilibrium equation given by

(5) $$\frac{\partial }{\partial x_{j}}\left(C_{ij}\frac{\partial u_{j}}{\partial x_{i}}\right)+f_{i}=\rho \frac{\partial^{2}u_{i}}{\partial t^{2}}\;$$

with the boundary conditions, $u=u_{g}$ on $\Gamma_{u}$ and $\frac{\partial u}{\partial x_{i}}=f_{s}$ on $\Gamma_{j}$. Here, *u* is the displacements field, $f_{i}$—the body force per volume (zero in this study), $f_{s}$—the surface traction per area, $C_{ij}$—material stiffness matrix, ρ—the material density, $u_{g}$—prescribed displacements. Moreover, the strains are given as the sum of elastic, thermal, and plastic stresses $\varepsilon_{ij}^{e}$, $\varepsilon_{ij}^{t}$, $\varepsilon_{ij}^{p}$, respectively.

(6) $$\varepsilon_{ij}=\frac{\partial u_{i}}{\partial x_{j}}=\varepsilon_{ij}^{e}+\varepsilon_{ij}^{t}+\varepsilon_{ij}^{p}\;$$

For the structural analysis, no surface traction was imposed on the part. However, a fully constrained displacement boundary condition was imposed, as shown in Figure 2b. These supports will be deleted during the spring-back phase of the simulation and will be discussed later. To avoid rigid body translation at this stage, one vertex at the center of the full part was also fully constrained for the duration of the whole simulation.

**Figure 3:**
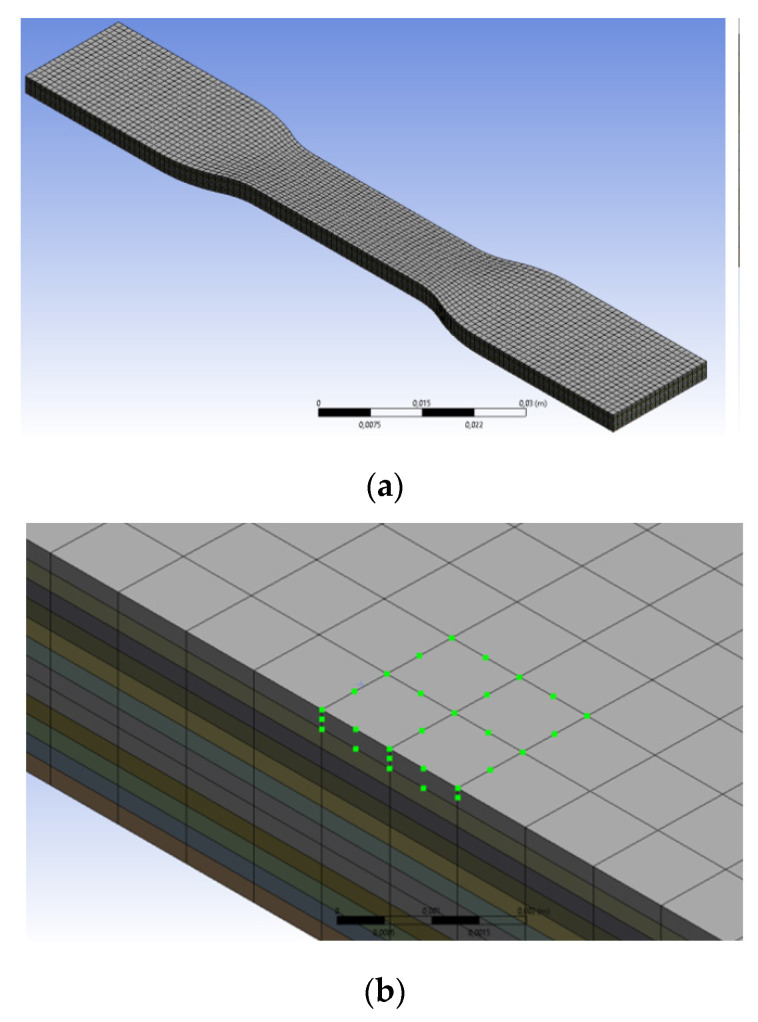
(**a**) General mesh of a body. (**b**) Second-order elements.

To discretize the model, the structured hex mesh was used, as shown in Figure 3a. The order of the mesh is two, meaning that there is a mid-side node on each edge of an element, as shown in Figure 3b. This allows using second-order shape functions, alleviates shear locking, and increases the accuracy of the solution for a given number of elements. The maximum size of the mesh is 1 mm along the x and z axes. A second-order interpolation was used and therefore, there were three nodes per element edge and the distance between two nodes is comparable with one road-width of the deposited filament. Along the *y*-axis, the size of a mesh is equal to the height of the layer.

The deposition of the molten plastic was modeled using the element birth and death method. In the following method, different elements can be activated at different time steps, and the part topology is updated according to the activation algorithm [21].

**Figure 4:**
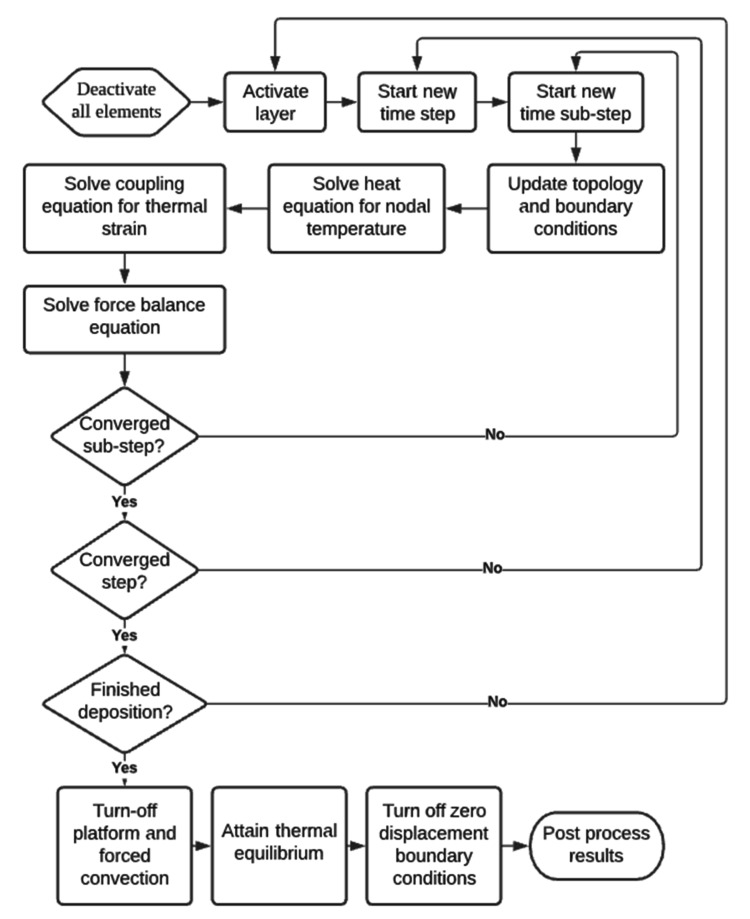
Algorithm used to perform simulations.

The approach utilized for our development is represented in Figure 4. First, the elements were deactivated except for the platform. Starting from the time step 1, the layers were activated one by one and left for cooling. Simultaneously, after each time sub-step within a time step, the thermal analysis was conducted first according to Equations (1)–(3). Then the thermal result was used to calculate thermal loading using Equation (4), and it was used as input for equilibrium Equations (5)–(6). The time sub-step incremented, and the equations were solved again until the whole step was resolved. After one layer was resolved entirely, the next layer was activated. After all the layers are activated, the platform’s temperature boundary condition is turned off, and it is left for cooling until it reaches equilibrium with the environment. Afterward, the part detachment is performed, and due to the thermal loading and constrained shrinkage, the part warps and deformations are obtained.

The material used in these simulations is Acrylonitrile Butadiene Styrene (ABS), and the material constants from Equations (1)–(6) are listed in Table 1. Other properties: heat capacity, heat transfer coefficient, Young’s modulus, and yield stress were set as temperature-dependent, and their variation was obtained from [25]. For this simulation, an elastic perfectly plastic hardening model was assumed. This assumption is in agreement with the findings of [33]. To avoid the convergence problem, the secant modulus in the plastic region was set to 10% of young’s modulus at the corresponding temperature.

### 2.2. Experimental Setup

**Figure 5:**
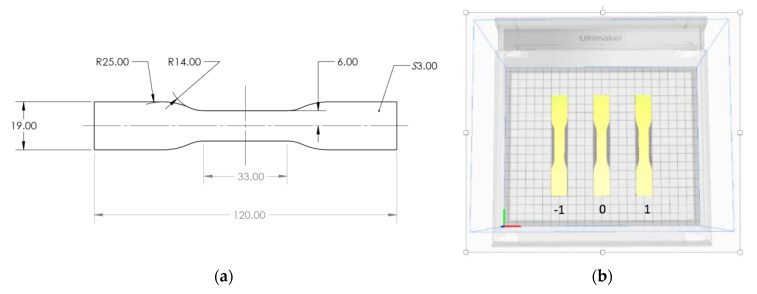
(**a**) The geometry of the sample. (**b**) The position of the samples during printing.

The samples were printed using the Ultimaker S3 printer (Ultimaker B.V., Utrecht, The Netherlands), which has a dual extrusion print head and a nozzle of 0.4 mm diameter. The geometry of the samples is shown in Figure 5. It was sliced in Ultimaker Cura software where extrusion temperature, bed temperature, and layer thickness were set individually for each experimental run according to the Taguchi orthogonal array (Table 2 and Table 3), while other parameters were not changed throughout the experiments and are shown in Table 4. The ABS filaments (Bestfilament”, Tomsk, Russia) were 2.85 mm in diameter. Three samples were printed for each experimental run resulting in 27 samples in total. Depending on the position, three samples were labeled such that the sample in the middle was denoted as “0” and the samples to the left and right of it were labeled “−1” and “1”, respectively (Figure 5b).

Before printing, the surface of the building platform was cleaned with the ethanol solution. To avoid excessive adhesion to the platform surface and subsequent damaging removal, no glue was applied. However, without glue, the samples were displaced from the specified positions by the movement of the nozzle and severely warped, which caused the nozzle to scratch the surface of the samples. Moreover, this scraping could damage the nozzle. Hence, brims were added to samples. After printing was completed, the samples were allowed to cool, then they were carefully removed from the platform and the brims were cut off. The platform was cleaned for the next experimental run and the procedure described above was repeated. The samples were then measured using a digital caliper.

**Figure 6:**
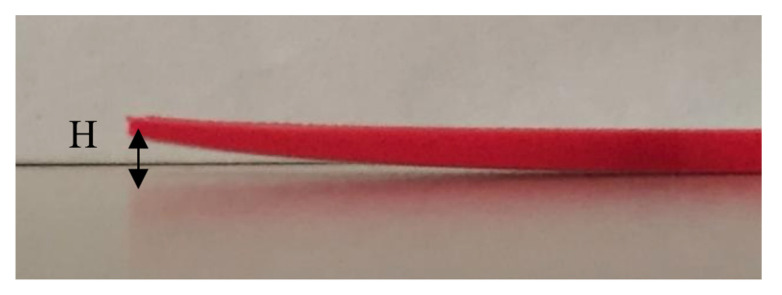
The warped edge of a sample.

Each sample was measured three times. Then the values were averaged. The parameter that denotes warpage was labeled as “H” and is shown in Figure 6.

## 3. Simulations and Experimental Results

**Figure 7:**
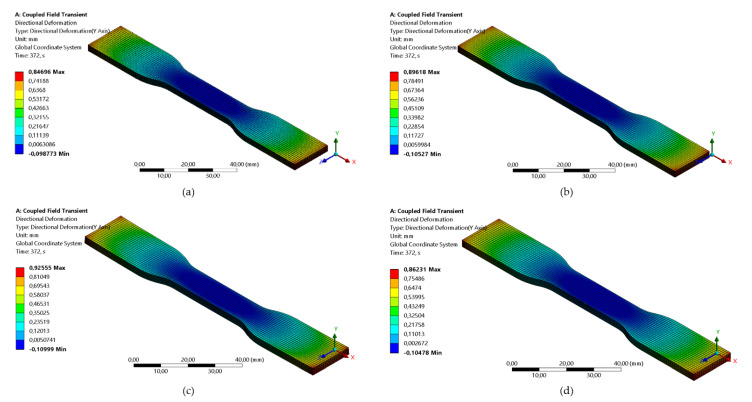

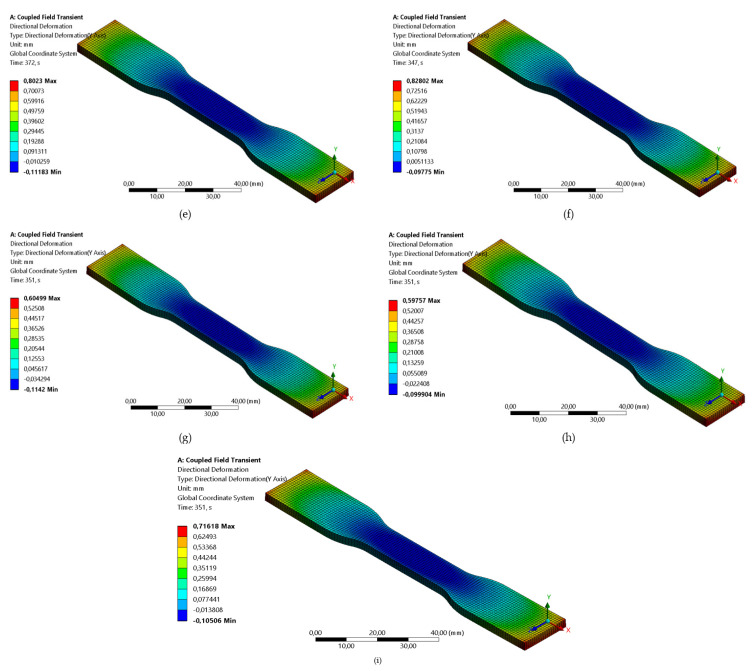
Directional deformation along the *y*-axis for run: (**a**) 1; (**b**) 2; (**c**) 3; (**d**) 4; (**e**) 5; (**f**) 6; (**g**) 7; (**h**) 8; (**i**) 9.

The simulations were run, parts were manufactured, and warpage was measured according to the procedure. Figure 7 shows the deformation of the part just after the removal of the supports. It is seen that the part warps and the maximum deformation is expected at the corners of the part. There is also a shrinkage center at the geometrical center of a part. The deformations close to it are low, which was also observed by [15]. Thus, the part attains the shape of the bowl. The reason for this pattern is the shrinkage of the part during cooling. Due to the shrinkage, internal forces are generated within a constrained part. These forces cause internal moments, and after the removal of the part, they cause warping [18,20].

**Figure 8:**
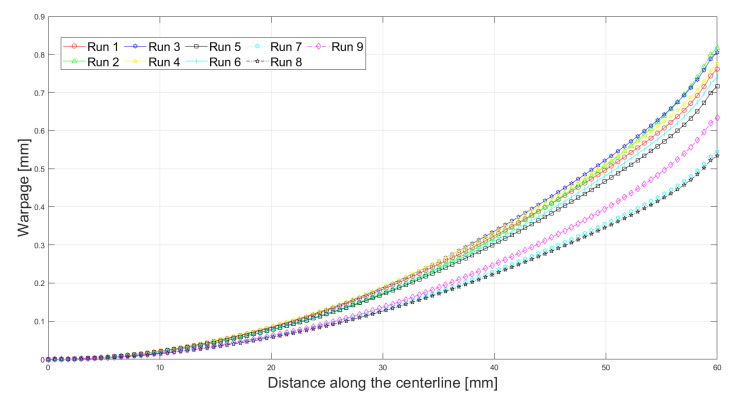
Warpage along the central half-length.

**Figure 9:**
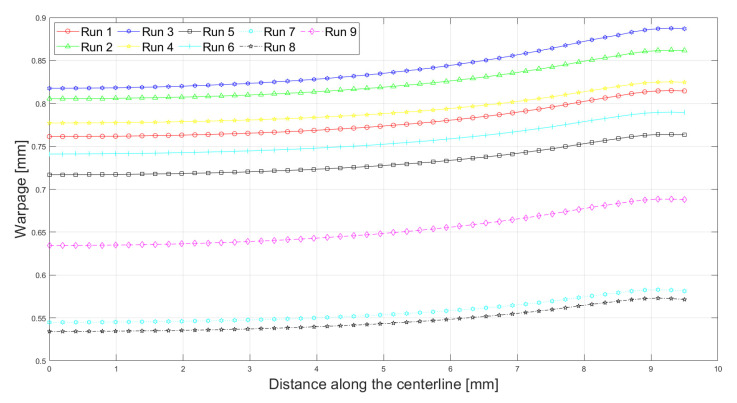
Warpage along the central half-width.

Figure 8 and Figure 9 show the warpage deformation along the central half-length and utmost half-width. It is seen that the warpage progresses along the length and width. At the center of the part, zero warpage is expected, while close to the end it attains maximum value. In addition, warpage along the length increases more compared to the width dimension. Thus, for longer dimensions, the warpage is larger. Similar findings were also observed by [20].

**Figure 10:**
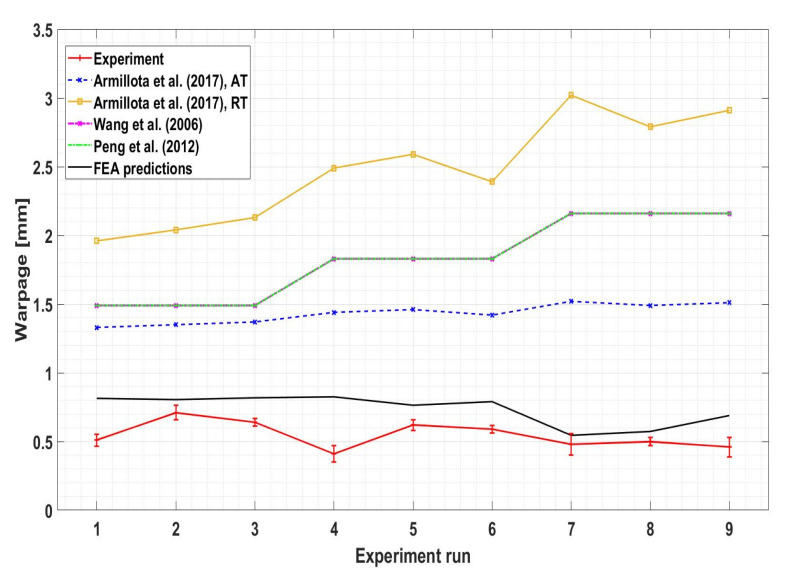
Comparison of FEA and analytical predictions with experimental results for warpage.

In order to compare the experimental results, numerical and several published analytical models, all the results were plotted, as shown in Figure 10. In addition, a comparison of experimental results and numerical predictions are listed in Table 5. Several analytical models were used to calculate warpage as the function of printing conditions. The results obtained using models developed by [17,19] were identical, as seen from the figure. This happened because they used similar principles (equilibrium) and assumptions (elastic loading at room temperature) in the derivations of their models. Note that warpage was predicted twice by Armillota et al. [20] using material properties at the room temperature (RT) conditions and the average temperature (AT) of the range. The latter model offered the best predictions for warpage among the given analytical models so far. The reason for this might be the inclusion of the yielding and layer reheating [21].

From Table 5, the predictions of the finite element method were found to be close to the experimental results in some simulations but diverged in others. The model fits the results well for Runs 2, 5, 7 and 8. However, for Run 4, the discrepancy between Finite Element prediction and experimental measurement is high. The reasons for this might be the assumptions employed in Finite Element modeling and human errors during the measurements. Indeed, from Figure 10, it can be noticed that 410 μm of warpage measured during the experiment is abnormally low compared to other printings with similar process parameters. It can be noted that the predictive capability of the model becomes better at higher levels of the layer thickness. These findings may be supported with the aid of surface chemistry and roughness. It is reported that decreased coating weight generates higher hydrophobicity and surface roughness while thick layers come with fewer empty spaces between the layers, resulting in a reduced hydrophobic effect. In addition, thin layers of filament are most likely to retain the intrinsic unevenness of the surface [34,35]. Some studies reported on the extensive hydrophobic nature of thin coating related to the higher surface roughness [36], while other studies [37] suggested decreased surface roughness as well as hydrophobicity due to filled up voids and formation of large aggregate in the case of multiple layers. However, this hypothesis needs to be further investigated.

It can also be noticed that the values obtained by the proposed model are consistently higher than the experimental results. Similar overprediction was obtained by [25]. Normally, the strain energy of the approximate FE solution is always not greater than that of the exact solution [38], and hence predicted deformations should be lower than measured. This discrepancy might be explained by the fact that in the current model, creeping of the part was not included in the calculation. Due to heating from the printing bed and nozzle, the printed part is always heated during the building process. The creep rate of ABS is significantly higher at elevated temperatures [39]. Hence, because of the action of the adhesion of part to the platform, which acts in the opposite direction of the warpage, the part experiences severe deformation and straightens. Because of neglecting this effect in the Finite Element model, results obtained using FEM are larger than those in the actual experiments.

## 4. Taguchi Optimization

The Taguchi method can help to design experiments to study the effects of process parameters on response parameters. In addition, it allows reducing the number of experimental runs without resorting to complicated calculations. In this study, the process parameters are layer thickness, extrusion, and bed temperature, while warpage was chosen as a response parameter. It is desired to reduce the warping of the samples. Hence, the smaller-is-better approach was used. To analyze the effects of the process parameters on the warpage, the S/N ratios need to be calculated. Equation (7) is used to calculate η (S/N ratio) for the smaller-is-better approach in Taguchi analyses, where σ, $Y_{avg}$, and $Y_{0}$ are variance, average, and target value, respectively. In this study, the target value is 0.

(7) $$\eta =-10\log\left(\sigma^{2}+{\left(Y_{avg}-Y_{0}\right)}^{2}\right)\;$$

(8) $$SST=\overset{N}{\underset{i=1}{\sum }}\left(\eta_{i}-\bar{\eta }\right)$$

(9) $$SS_{j}=\overset{L}{\underset{i=1}{\sum }}\left(\eta_{ji}-\bar{\eta }\right)\;$$

(10) $$MS_{j}=\frac{SS_{j}}{DOF_{j}}\;$$

(11) $$F_{j}=\frac{MS_{j}}{MS_{e}}\;$$

For ANOVA analysis, SST, $SS_{j}$, $MS_{j}$, $F_{j}$ values were calculated using Equations (8)–(11), where SST is the total sum of squares and $\bar{\eta }\;$ is called the average of S/N ratios of N number of experiments. $SS_{j}$ is called the sum of squared deviations of the jth factor and L is the level of that factor. $MS_{j}$ and $DOF_{j}\;$ are called the variance and the degree of freedom of the jth factor, respectively. $F_{j}$ is the F-value of the jth factor and is calculated by dividing $MS_{j}$ by the error’s variance ($MS_{e}$).

**Figure 11:**
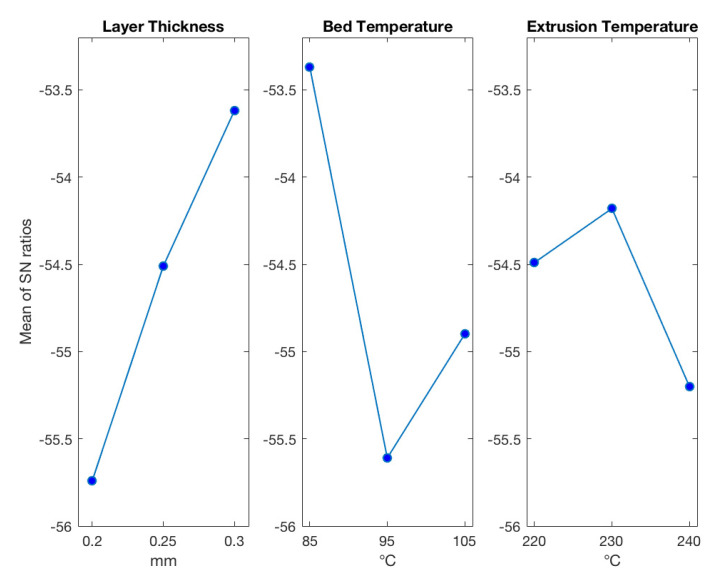
Experimentally derived results for warpage.

The results of calculations can be seen in Table 6 and Table 7 for experimental and FEM predicted values of H, respectively. The larger values of S/N ratios indicate the optimum level of the parameter. Taguchi optimization from experimental results showed that for minimum warpage deviation layer thickness, bed temperature and extrusion temperature should be at levels 3, 1, and 2, respectively (Figure 11). ANOVA analysis can show the statistical significance of factors if the *p*-value is less than 0.05. The *p*-values from experimental H analyses were 0.272, 0.243, 0.607 for layer thickness, bed temperature, and extrusion temperature, respectively (Table 8).

**Figure 12:**
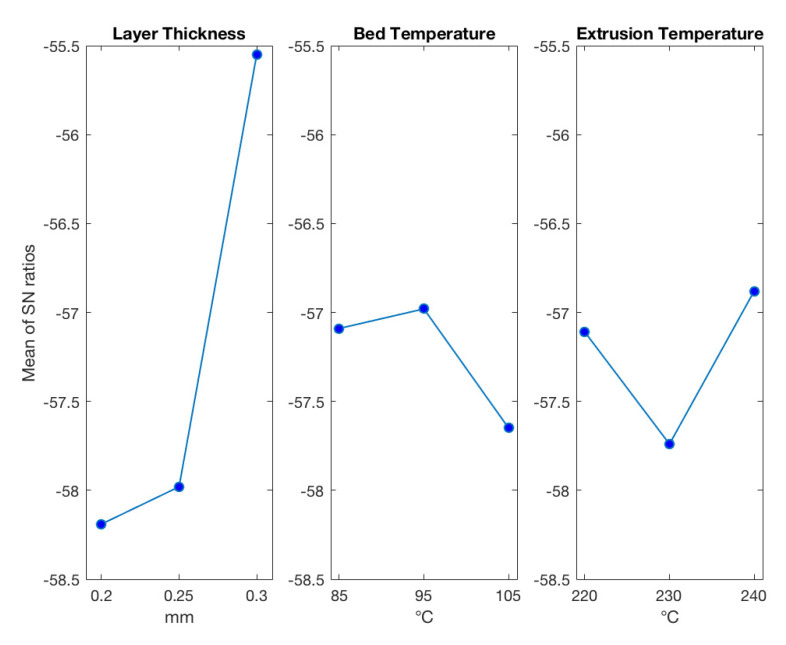
FEM results for warpage.

Taguchi optimization of warpage deviations using FEM results are shown for minimum warpage when levels of input parameters are as follows, 3, 2, and 3 (Figure 12). The *p*-values from ANOVA analyses were 0.042, 0.419, 0.343 for layer thickness, bed temperature, and extrusion temperature, respectively (Table 9).

According to the optimization based on experimental results (Figure 11 and Table 7), layer thickness and bed temperature are the most significant factors affecting the warpage of the part. Layer thickness and bed temperature have a contribution of approximately 36% and 42% to the final warpage. Additionally, the dependence of the warpage on the layer thickness is monotonic, and with an increase in the layer thickness, the warpage is minimized. On the other hand, the dependence of the warpage on the bed temperature is not monotonic.

Similarly, according to the optimization based on simulation results (Figure 12 and Table 9), layer thickness solely has the largest impact on the warpage. Its contribution is about 83.6% Again, simulation results show the inverse monotonic correlation between warpage and layer thickness, which agrees with experimental results. Similar results were also observed in other works [13,14,15,16].

To verify the results, the optimum levels of the process parameters were set, and the samples were printed using those parameters. The measured values of the warpage can be seen in Table 10. Optimum process parameters based on the results of the FE simulations lead to a part with a slightly smaller warpage value of 310 microns. At the same time, optimum process parameters based on the experimental results produce a part with a warpage equal to 320 microns. Nevertheless, both samples yield to lower warpage compared to the results from nine runs shown in Table 5.

## 5. Model Validation for Multilaterals

The application of FEM using ANSYS ^®^ (ANSYS 2020R2, Canonsburg, Pennsylvania) was further extended to predict the warpage of FDM printed multi-material parts. In this study, HIPS (High Impact Polystyrene, Bestfilament”, Tomsk, Russia) thermoplastic was used in different combinations with ABS (Acrylonitrile Butadiene Styrene (Bestfilament”, Tomsk, Russia) material because of their better compatibility and uniformity when printed on top of each other [40]. As in the case of pure ABS part, a bilinear plastic model was used for HIPS Material. Both constant and transient material properties for HIPS material were based on the secondary findings, as shown in Table 11.

The effect of material combinations on the warpage of printed multi-material parts was studied using a numerical study. The following material combinations were studied both numerically and experimentally:

Alternating specimen (AA HH AA HH AA HH)

Sandwich specimen (AAA HHHH AAA)

**Figure 13:**
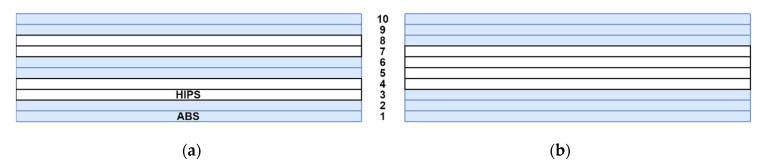
Multi-material combination schematics: (**a**) alternating specimen, (**b**) sandwich specimen.

**Figure 14:**
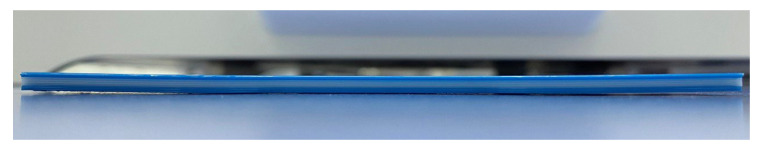
Printed multi-material sandwich specimen.

Note that HH stands for the two layers of the HIPS material, whereas AAA denotes the three layers of the ABS plastic (see Figure 13). Figure 14 shows the illustration of a printed multi-material sandwich specimen (AAA HHHH AAA).

**Figure 15:**
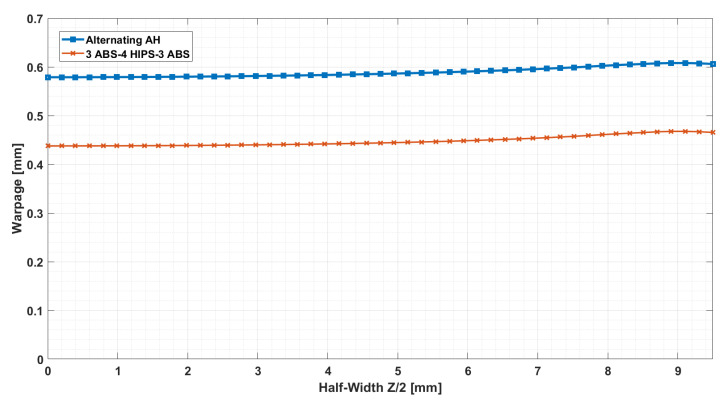
Warpage of multi-material parts along the half-width.

The same process parameters (0.3 mm layer thickness, 95 °C platform temperature, and 240 °C nozzle temperature) were used for both numerical and experimental studies. The numerical simulation result for the part warpage is presented in Figure 15.

Table 12 provides detailed values of the numerical and experimental findings in terms of printed part warpage. The same material combinations were printed using a commercial Ultimaker S3 FDM printer. HIPS and ABS thermoplastics were obtained from the “Best filaments” manufacturer.

It can be noted that the FEM predicted values for the part warpage are bigger than the corresponding experimental findings. This implies that FEM overestimates the dimensional deviation of FDM printed parts. The same finding was stated in other literature [25]. The relative percentage error between the numerical and experimental warpage results for alternating and sandwich specimens are 1.4% and 9.5%, respectively (see Table 12). In this study, all the material properties were obtained from the existing literature and therefore might not be the same as the utilized thermoplastics. This can be considered as a feasible reason for the discrepancy between the numerical and experimental results. For example, the warpage prediction using FEM was shown to be linearly dependent on the CTE of the assigned material [42]. Therefore, the accuracy of numerical simulation in predicting the warpage of FDM printed parts can be enhanced by implementing the exact material properties as an input.

## 6. Conclusions

In this study, the FDM printing process was simulated to predict the warping deformation of the printed samples made from ABS only and from altering ABS-HIPS combinations (multi-material parts). The results were compared with analytical models from the literature and with the experimental results. The FEA model showed that samples warp in a bowl-like shape, which was also observed on experimentally printed parts. The predictions of the FEA model are closer to the actual warpage at higher values of the layer thickness. From this investigation, the following conclusions were observed:

- Both simulated and experimental results showed that the warpage decreases with increasing layer thickness.
- With regards to the analytical models, all models predicted much higher warping deformation compared to the experimental values and their respective numerical approximations. It was observed that using a model developed by Armillota et al. [20], calculated warpage values became more in line with experimental data when the average temperature was used instead of room temperature.
- In all analytical models and the developed FEA model, the warpage was overestimated. On the other hand, the FE results for displacement should be lower because the stiffness matrix obtained through the Finite Element solution is stiffer. This might happen because the assumptions employed in the FE modeling for the simplicity effect of the creep were not ignored.
- Regarding simulations of multi-material parts, the relative percentage error between the numerical and experimental warpage results for alternating and sandwich specimens are 1.4% and 9.5%, respectively.

## Figures and Tables

**Table 1:** Constant material properties.

**Density (** ρ **)**	1040 $\mathrm{kg}/m^{3}$	Poisson Ratio (v)	0.38
**Glass transition temperature** ** (** $T_{g}$ **)**	105 °C	Thermal expansion (α)	9 × 10^−5^/°C

**Table 2:** Factors and levels.

Factor	Symbol	Level			Unit
		1	2	3	
Layer thickness	A	0.1	0.2	0.3	mm
Bed temperature	B	85	95	105	°C
Nozzle temperature	C	220	230	240	°C

**Table 3:** L9 orthogonal array.

Experimental Run №	A (mm)	B (°C)	C (°C)
1	0.2	85	220
2	0.2	95	230
3	0.2	105	240
4	0.25	85	230
5	0.25	95	240
6	0.25	105	220
7	0.3	85	240
8	0.3	95	220
9	0.3	105	230

**Table 4:** Default Printing factors.

Factor	Value	Unit
Wall thickness	1.3	mm
Infill density	100	%
Infill pattern	Rectilinear	-
Print speed	55	mm/s
Fan speed	2	%

**Table 5:** Comparison of simulation predictions with experimental results for warpage.

Run №	Layer Thickness (mm)	Bed Temperature (°C)	Extrusion Temperature (°C)	Experimental H (µm)	Predicted H (µm)	Error %
1	0.2	85	220	506	814	60.9
2	0.2	95	230	709	805	12.8
3	0.2	105	240	639	818	28.0
4	0.25	85	230	414	825	99.3
5	0.25	95	240	617	764	23.8
6	0.25	105	220	588	790	34.4
7	0.3	85	240	483	545	12.8
8	0.3	95	220	501	573	14.3
9	0.3	105	230	457	689	50.8

**Table 6:** S/N ratios response table for experimental H.

Level	Layer Thickness (mm)	Bed Temperature (°C)	Nozzle Temperature (°C)
1	−55.74	−53.37	−54.49
2	−54.51	−55.61	−54.18
3	−53.62	−54.90	−55.20
Delta	2.11	2.24	1.01
Rank	2	1	3

**Table 7:** S/N ratios response table for FEM predicted H.

Level	Layer Thickness	Bed Temperature	Nozzle Temperature
1	−58.19	−57.09	−57.11
2	−57.98	−56.98	−57.74
3	−55.55	−57.65	−56.88
Delta	2.64	0.67	0.86
Rank	1	3	2

**Table 8:** ANOVA table for the warpage optimization based on experimental results.

Source	DOF	SS	MS	F	*p*	Contribution
A	2	6.713	3.356	2.675	0.272	35.961
B	2	7.818	3.909	3.116	0.243	41.885
C	2	1.626	0.813	0.648	0.607	8.711
Error	2	2.509	1.255	-	-	13.443
Total	8	18.666	-	-	-	100

**Table 9:** ANOVA table for FEM predicted H.

Source	DOF	SS	MS	F	*p*	Contribution
A	2	12.911	6.455	22.819	0.042	83.60
B	2	0.784	0.392	1.385	0.419	5.07
C	2	1.184	0.592	2.092	0.343	7.66
Error	2	0.566	0.283	-	-	3.66
Total	8	15.444	-	-	-	100

**Table 10:** Warpage at optimum parameters.

	Factor and Levels	Measured H (μm)
Experiment	$A_{3}B_{1}C_{2}$	320
Simulation	$A_{3}B_{2}C_{3}$	310

**Table 11:** Material properties for HIPS material.

Property	Value	Source
Glass transition temperature (°C)	100	[40]
Density (kg/m^3^)	1048	[41]
CTE (Coefficient of thermal expansion) (1/°C)	6.7 × 10^–5^	[42]
Thermal conductivity (W/mK)	Transient	[41]
Specific Heat (J/kgK)	Transient	[41]
Elastic Modulus (MPa)	Transient	[43]
Yield Strength (MPa)	Transient	[43]

**Table 12:** Numerical simulation and experimental results.

Material Combination	Warpage (µm)		Error (%)
	FEM	Experimental	
Alternating specimen (AA HH AA HH AA HH)	607.93	616.67	1.4
Sandwich specimen (AAA HHHH AAA)	467.63	516.67	9.5

## Data Availability

The data presented in this study are available on request from the corresponding author.

## Nomenclature

Symbol	Meaning
c	Specific Heat
T	Temperature
t	Time
L	Length
A	Coefficient of Thermal Expansion
ρ	Material Density
k	Coefficient of Thermal Conductivity
q	Internal Heat Generation per unit Volume
$\Gamma_{u}$	Dirichlet Boundary
$\Gamma_{j}$	Neuman Boundary
$n_{i}$	Unit normal vector
$T_{0}$	Initial Temperature
$q_{c}$	Convective Heat Flux
h	Convective Heat Transfer Temperature
$T_{c}$	Temperature of the surrounding medium
${\mathrm{Nu}}_{L}$	Nusselt Number
$\varepsilon_{ij}^{t}$	Thermal Strain
$\varepsilon_{ij}^{e}$	Elastic Strain
$\varepsilon_{ij}^{p}$	Plastic Strain
$u_{j}$	Displacement Vector
$C_{\mathrm{ij}}$	Material Stiffness Matrix
$f_{i}$	Body Force
$f_{s}$	Surface Force
η	S/N Ratio
σ	Variance
$Y_{avg}$	Average Response
$Y_{0}$	Target Response
SST	Total Sum of the Squares
$SS_{j}$	Sum of Squared Deviations of the jth Factor
$MS_{j}$	Variance of the jth factor
$DOF_{j}$	Degree of Freedom of the jth factor
$F_{j}$	F-value of the jth factor
